# Arthroscopy-Assisted Core Decompression Combined with Octacalcium Phosphate/Gelatin Composite Implantation for Osteonecrosis of the Femoral Head: A Study Protocol for a Single-Center Externally Controlled Trial

**DOI:** 10.3390/medsci14030339

**Published:** 2026-06-23

**Authors:** Hidetatsu Tanaka, Kazuyoshi Baba, Ryuichi Kanabuchi, Yasuaki Kuriyama, Hiroki Kawamata, Hideki Fukuchi, Yu Mori, Toshimi Aizawa

**Affiliations:** Department of Orthopaedic Surgery, Tohoku University Graduate School of Medicine, 1-1 Seiryo-machi, Aoba-Ku, Sendai 980-8574, Miyagi, Japan; kazuyoshi.baba.e3@tohoku.ac.jp (K.B.); ryuichi.kanabuchi.b8@tohoku.ac.jp (R.K.); rev.27.chemt@gmail.com (Y.K.); hiroki.kawamata.e4@tohoku.ac.jp (H.K.); hideki.fukuchi.a3@tohoku.ac.jp (H.F.); yu.mori.c4@tohoku.ac.jp (Y.M.); toshimi.aizawa.a5@tohoku.ac.jp (T.A.)

**Keywords:** osteonecrosis of the femoral head, joint-preserving surgery, core decompression, osteoconductive biomaterials, arthroscopy-assisted surgery

## Abstract

**Background/Objectives:** Osteonecrosis of the femoral head is a progressive disease that frequently leads to femoral head collapse and secondary osteoarthritis. Although total hip arthroplasty provides reliable outcomes, its use in younger patients is limited due to concerns regarding implant longevity. Joint-preserving procedures such as core decompression have been widely used; however, their efficacy remains controversial. This study aims to evaluate a combined approach using arthroscopy-assisted core decompression and an osteoconductive bone substitute. **Methods:** This study is designed as a single-center, externally controlled trial conducted at Tohoku University Hospital. Patients with osteonecrosis of the femoral head (Japanese Investigation Committee Stage 1–3B, Type B–C2) will undergo arthroscopy-assisted core decompression combined with octacalcium phosphate/gelatin composite implantation. A total of 25 patients will be prospectively enrolled. Outcomes will be compared with a propensity score-matched historical control cohort. The primary outcome is disease progression within 1 year, defined as radiographic progression or conversion to total hip arthroplasty. Secondary outcomes include radiographic changes, clinical outcomes, and bone remodeling assessed by computed tomography. **Expected Results:** This study is expected to provide preliminary clinical evidence regarding the feasibility and potential effectiveness of arthroscopy-assisted core decompression combined with octacalcium phosphate/gelatin composite implantation for osteonecrosis of the femoral head. The intervention may promote bone remodeling and contribute to the prevention of femoral head collapse. **Conclusions:** The findings of this study may contribute to the development of improved minimally invasive joint-preserving treatment strategies for osteonecrosis of the femoral head and provide a basis for future large-scale clinical trials.

## 1. Introduction

Osteonecrosis of the femoral head (ONFH) is a common hip disorder characterized by femoral head collapse, which can lead to secondary osteoarthritis, resulting in severe hip pain and functional limitations [1]. In most cases, intraosseous necrosis has already occurred at the time of diagnosis, making early detection difficult, and patients are often asymptomatic before femoral head collapse, with spontaneous reduction in the necrotic area being rare and larger lesions more likely to progress to collapse [1,2]. A national epidemiological survey conducted in 2004 estimated that approximately 11,400 patients with ONFH were treated annually in Japan, with approximately 2200 new cases diagnosed each year [3]. Once femoral head collapse occurs, patients develop pain and gait disturbance, ultimately leading to irreversible deterioration of hip joint function, and many patients with ONFH require surgical treatment, with more than half undergoing surgery within nine months of diagnosis [4]. Total hip arthroplasty (THA) is indicated in advanced stages with substantial joint destruction and provides excellent pain relief and functional outcomes; however, complications such as infection and dislocation can occur, and long-term mechanical loosening may necessitate revision surgery [1,5,6]. Moreover, several studies have suggested that patients with ONFH may experience higher complication and revision rates after THA compared with those with primary osteoarthritis [7,8,9]. Because ONFH often affects relatively younger patients, the indication for THA should be considered carefully, and joint-preserving procedures are preferred before substantial joint destruction occurs [5,10]. Femoral osteotomy procedures, such as transtrochanteric rotational osteotomy and curved varus osteotomy, have been widely performed in Japan to transfer the viable portion of the femoral head to the weight-bearing area while shifting the necrotic region away from load-bearing zones [11,12]. However, these procedures are highly invasive, involving substantial disruption to bone, musculotendinous structures, and the joint capsule, and their utilization has shown a decreasing trend based on a Japanese nationwide database study [13].

Core decompression is a less invasive joint-preserving option. This procedure involves drilling into the necrotic area from the lateral femoral cortex to reduce intraosseous pressure and promote revascularization [10,14]. However, according to the Japanese Orthopaedic Association Guidelines for Osteonecrosis of the Femoral Head 2019, no consensus has been reached regarding the efficacy of core decompression (Recommendation grade 5, evidence level D), reflecting the limited and inconsistent clinical evidence as well as variability in reported outcomes across studies [11]. Recent systematic reviews and meta-analyses have suggested that core decompression may delay disease progression in selected patients with early-stage ONFH. Nevertheless, substantial heterogeneity among surgical techniques, adjunctive procedures, and patient populations precludes definitive conclusions regarding its efficacy [15,16]. Surgical treatments combined with core decompression include cell-based and growth factor therapies, such as bone marrow-derived mononuclear cells [17,18,19,20], bone marrow-derived mesenchymal stem cells [21], platelet-rich plasma [22], bone morphogenetic proteins (BMP-2 and BMP-7) used in combination with bone grafting [23,24,25], and fibroblast growth factor-2 (FGF-2) combined with core decompression [26]. Alternatively, core decompression combined with artificial bone grafting, such as β-tricalcium phosphate (β-TCP), has been reported, with favorable clinical outcomes [27,28]. Since femoral head collapse creates structural voids and instability within the necrotic region, filling these defects is considered biomechanically reasonable. β-TCP grafting has been reported to achieve outcomes comparable to vascularized fibular grafting [29] and to be less invasive than allogeneic bone grafting while remaining effective [30]. Octacalcium phosphate/gelatin composite (OCP/Gel) has demonstrated superior osteoconductivity compared with β-TCP mainly in preclinical studies, including animal experiments and in vitro investigations [31,32,33,34,35,36]. However, clinical evidence in humans remains limited. Recent transcriptomic analyses using comprehensive RNA sequencing have revealed that OCP/Gel exerts pro-angiogenic effects, suggesting that this material may enhance vascularization within the necrotic lesion and thereby contribute to bone regeneration [37]. It has been commercialized and may enhance bone regeneration, making it a promising material for defect filling. In addition, in pre-collapse stages, OCP/Gel may promote regeneration of the necrotic area and potentially prevent subsequent femoral head collapse. Therefore, this material may provide both regenerative and preventive benefits in the treatment of ONFH. Hip arthroscopy has been explored as a minimally invasive option for the management of ONFH, particularly in early-stage disease or in combination with core decompression; however, its role remains limited, and the current evidence is insufficient to establish its efficacy as a standalone treatment [38,39].

The aim of this study is to evaluate the efficacy and safety of arthroscopy-assisted core decompression combined with OCP/Gel implantation for patients with osteonecrosis of the femoral head. Specifically, this study will assess its potential to promote bone regeneration and prevent femoral head collapse, while enabling the simultaneous evaluation and treatment of intra-articular pathology.

## 2. Materials and Methods

### 2.1. Study Design

**Figure 1:**
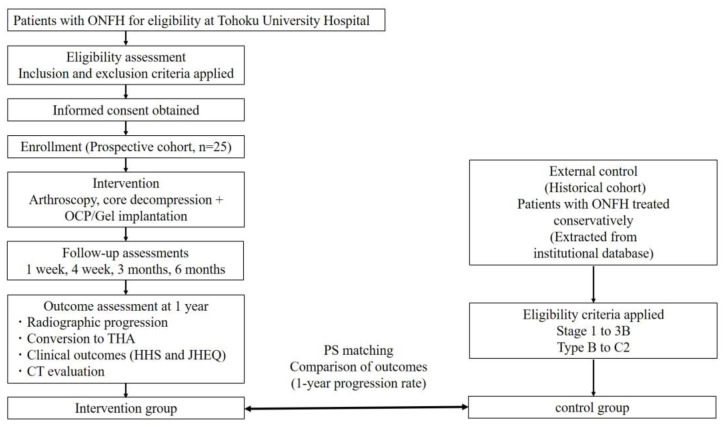
Study flowchart. Patients with ONFH are prospectively enrolled and undergo arthroscopy-assisted core decompression combined with OCP/Gel implantation. Outcomes are compared with a propensity score–matched historical control cohort.

This study is a single-center, externally controlled trial. Patients undergoing artificial bone grafting will be compared with a historical cohort of patients with ONFH treated conservatively. Propensity score matching will be used to adjust for baseline differences. The flowchart of the study is shown in Figure 1.

This study was approved by the Tohoku Certified Review Board of Tohoku University (approval number: CRB2200003, approval date: 30 April 2025) and will be conducted in accordance with the Declaration of Helsinki. Written informed consent will be obtained from all participants in the prospective intervention cohort prior to enrolment. For the historical control cohort, the requirement for informed consent was waived by the ethics committee due to the use of anonymized retrospective data.

A randomized controlled trial was not feasible for this study due to the low incidence of ONFH, the novel nature of the intervention with no established efficacy data, and ethical considerations regarding withholding a potentially beneficial joint-preserving treatment from younger patients at risk of femoral head collapse. Therefore, this study was designed as a single-center, externally controlled pilot trial.

The trial has been approved by the institutional review board of the Tohoku Certified Review Board of Tohoku University (Ministry of Health, Labor and Welfare Certified Clinical Research Review Board, Tohoku University; approval number: CRB2200003) and is registered at jRCT (Japan Registry of Clinical Trial; jRCT1022250003; registration date: 21 May 2025, URL: https://jrct.mhlw.go.jp/re/reports/detail/10819122 (accessed on: 21 May 2025).

### 2.2. Population

We will include 25 patients with ONFH. As an external control, historical data will be extracted from patients previously diagnosed with ONFH and treated conservatively at Tohoku University Hospital. Eligible patients in the historical cohort must meet the following criteria: (1) diagnosis of ONFH based on radiographic and MRI findings at the time of initial presentation; (2) classification as Stage 1, 2, 3A, or 3B (excluding Stage 4) and Type B, C1, or C2 according to the criteria of the Japanese Investigation Committee (JIC) of Idiopathic Osteonecrosis of the Femoral Head [40]; (3) age ≥ 18 years at the time of diagnosis; and (4) no restriction on sex [41]. Eligible patients in the historical cohort will be selected to match the study population as closely as possible. Historical control data will be extracted from patients treated conservatively at Tohoku University Hospital between Jun 2005 and May 2021. During this period, radiographic and MRI-based diagnosis and staging were performed according to the Japanese Investigation Committee classification. Nevertheless, temporal changes in clinical practice and patient management may have influenced the outcomes and should be considered when interpreting the results.

### 2.3. Inclusion Criteria

Patients will be eligible if they meet the following criteria:

(1) Diagnosed with ONFH at Tohoku University Hospital
(2) Stage 1–3B and Type B–C2 according to the Japanese classification
(3) Age ≥ 18 years
(4) Provided written informed consent
(5) Surgery can be performed within 18 months after the initial diagnosis or worsening of symptoms.

No upper age limit is specified because the study aims to reflect real-world clinical practice. Most enrolled patients are expected to be relatively young to middle-aged adults, consistent with the epidemiology of ONFH.

### 2.4. Exclusion Criteria

Patients will be excluded if they:

(1) Have a history of hip surgery
(2) Are pregnant or breastfeeding
(3) Have ONFH secondary to trauma, tumor, or other specific diseases. Patients with steroid-induced or alcohol-related ONFH will be included because these etiologies represent common forms of non-traumatic ONFH.
(4) Have contraindications to artificial bone materials.

### 2.5. Intervention

All surgical procedures will be performed under general anesthesia using a traction table. First, hip arthroscopy will be conducted to assess intra-articular pathology, including acetabular cartilage, femoral head cartilage, and labral integrity. If a labral tear is identified, appropriate repair will be performed. Subsequently, core decompression will be performed under fluoroscopic guidance. A guidewire will be inserted into the necrotic lesion to confirm correct positioning. Core decompression will be performed using a cylindrical bone biopsy instrument. Next, OCP/Gel (Bricta^®^, Nipro, Osaka, Japan) will be transplanted into the necrotic cavity. OCP/Gel, which received regulatory approval in Japan in May 2024 and has been covered by national health insurance since October 2024, was used as the graft material in this study. The volume of graft material will be determined intraoperatively according to the extent of the lesion and the size of the cavity created during core decompression. Formal volumetric assessment is not planned in this pilot study. Because the graft material is radiolucent, intraoperative assessment of graft filling will be performed based on the insertion depth and position of the delivery instrument, as well as arthroscopic visualization. Finally, the joint will be inspected arthroscopically to confirm the absence of graft leakage. All procedures will be performed by experienced orthopedic surgeons familiar with hip arthroscopy and joint-preserving procedures for ONFH.

### 2.6. Evaluation Methods

**Primary outcome**

The primary outcome will be progression of ONFH within 1 year from baseline. Progression will be defined as the occurrence of either of the following events: (1) radiographic stage progression according to the classification system of the Japanese Investigation Committee of Idiopathic Osteonecrosis of the Femoral Head, or (2) conversion to total hip arthroplasty (THA). Baseline will be defined as the time of surgery in the intervention group and the time of initial diagnosis based on radiographic findings at Tohoku University Hospital in the historical control group. Radiographic stage progression will be assessed using anteroposterior radiographs obtained preoperatively and at 1 year. Collapse will be quantified by measuring the collapse length (mm), defined as the distance between the femoral head contour and a best-fit spherical outline on the femoral head. Radiographic evaluations, including assessment of disease progression and collapse length, will be performed independently by two orthopedic surgeons. Any discrepancies will be resolved by consensus. Because complete blinding to clinical information is difficult in this study, formal blinded assessment is not planned. In addition, formal assessment of interobserver agreement is not planned because of the exploratory nature of this pilot study.

**Secondary outcomes**

The secondary outcomes will include the following:

(1) Radiographic progression assessed at 3, 6, and 12 months using the same classification system
(2) Changes in collapse length (mm) measured on serial radiographs at baseline, 3, 6, and 12 months
(3) Clinical outcomes, including Harris Hip Score (HHS) and Japanese Orthopaedic Association Hip-Disease Evaluation Questionnaire (JHEQ), evaluated at baseline, 3 months, and 12 months
(4) Bone remodeling of the grafted area assessed using computed tomography (CT) before surgery and at 3 and 12 months postoperatively.

### 2.7. Data Collection

All data will be recorded electronically using standardized case report forms. The case report forms will include demographic data (age and sex), underlying conditions associated with ONFH, medication history, and medical history. In addition, data obtained during eligibility assessment will be collected, including clinical laboratory tests and imaging findings (X-ray and CT). These data will be used to assess baseline characteristics, clinical outcomes, and safety. Data accuracy will be ensured through regular monitoring and verification by the study investigators (Table 1).

### 2.8. The Study Procedure and Schedule

The study procedures and schedule are summarized in Table 1, in accordance with the SPIRIT recommendations. Eligible patients will be identified during outpatient visits. After a detailed explanation of the study, written informed consent will be obtained. Participants may withdraw their consent at any time without any consequences. Following consent, screening assessments will be performed within 1 month prior to surgery. Baseline data, including demographic characteristics (age, sex, height, and weight), medical history, and comorbidities, will be collected from medical records. Eligibility will be confirmed, and patient registration will be completed before surgery. All enrolled patients will undergo the surgical procedure as described above. Postoperative follow-up assessments will be conducted at 1 week, 4 weeks, 3 months, 6 months, and 12 months after surgery. Radiographic evaluation using X-rays will be performed at baseline and at each follow-up visit. Computed tomography (CT) will be performed at baseline and during follow-up to assess bone remodeling. Clinical laboratory tests will be conducted at baseline and during the early postoperative period. Vital signs, including blood pressure, pulse rate, body temperature, and respiratory rate, will be monitored during hospitalization and the immediate postoperative period. Patient-reported outcomes, including the HHS [42] and JHEQ [43], will be assessed at baseline, 3 months, and 12 months. Adverse events will be monitored throughout the study period regardless of their causal relationship with the intervention. Rehabilitation will be initiated during hospitalization and continued after discharge according to the patient’s weight-bearing status. Postoperative rehabilitation will generally be performed once daily during hospitalization and approximately once weekly until 6 weeks after surgery, with further adjustments as clinically indicated. Patients will remain non-weight-bearing for 3 weeks after surgery, followed by gradual progression to weight-bearing according to clinical symptoms and radiographic findings. Information on concomitant medications, including corticosteroids, immunosuppressive agents, and osteoporosis treatments, will be collected from baseline until the end of follow-up.

### 2.9. Data Management

All data will be collected and recorded using standardized electronic case report forms. Data entry will be performed by trained investigators and verified to ensure accuracy and completeness. Data quality will be maintained through regular monitoring and validation procedures. All patient information will be anonymized prior to analysis to ensure confidentiality. Access to the study database will be restricted to authorized study personnel only.

### 2.10. Statistical Analysis

As this study is designed as a pilot exploratory trial, a formal sample size calculation based on power analysis was not performed. A target sample size of 25 patients was determined based on the following considerations: (1) the rarity of ONFH, with an estimated annual incidence of approximately 2200 cases in Japan; (2) the novel nature of the intervention, for which no prior data are available to estimate effect size; and (3) the general recommendation that pilot studies should enroll at least 25 participants to provide a preliminary estimate of the effect size with sufficient precision for planning future confirmatory trials [44]. The findings of this study are intended to inform the design of future large-scale trials. Previous studies have reported that the progression rate of conservatively managed ONFH varies depending on disease stage and lesion size. The present pilot study is intended to provide preliminary estimates of progression rates and effect sizes, which will be used to inform sample size calculations for future confirmatory studies.

Continuous variables will be expressed as mean ± standard deviation or median (interquartile range), as appropriate, and categorical variables as frequencies and percentages. The primary outcome (progression of ONFH within 1 year) will be compared between the intervention group and the historical control group. To reduce confounding due to the non-randomized design, propensity score matching will be performed. The propensity score will be estimated using a logistic regression model including age, sex, body mass index, disease stage, disease type, and etiology of ONFH. Information regarding corticosteroid use, alcohol consumption, concomitant medications, and year of diagnosis will also be collected. Because of the limited sample size and exploratory nature of this pilot study, these variables will not be included in the primary propensity score model to avoid overfitting but will be described and considered when interpreting the results. Patients will be matched 1:1 using nearest-neighbor matching with a caliper width of 0.2 times the standard deviation of the propensity score. Patients for whom an adequate match cannot be identified will be excluded from the matched analysis. This may reduce the effective sample size and limit the generalizability of the findings. Covariate balance after matching will be assessed using standardized mean differences, with values < 0.1 indicating adequate balance. After matching, paired categorical variables, including the primary outcome, will be analyzed using McNemar’s test. Secondary outcomes will be analyzed descriptively and compared between groups using appropriate statistical methods. Missing data will be handled using complete case analysis. Because the historical control cohort is derived from institutional databases with routinely collected clinical and imaging records, the amount of missing data is expected to be minimal. Therefore, complete case analysis was considered appropriate for this exploratory study. Because the amount of missing data is expected to be minimal, formal sensitivity analyses or multiple imputation procedures are not planned. Alternative approaches to missing data will be considered in future confirmatory studies if necessary. All statistical analyses will be performed using JMP version 19.0 (SAS Institute, Cary, NC, USA). A two-sided *p*-value < 0.05 will be considered statistically significant.

### 2.11. Safety Assessment

All adverse events (AEs) occurring after surgery will be recorded regardless of causality, with pre-existing conditions excluded unless worsened. AEs will be collected from the time of surgery, with all procedure- and device-related events recorded within 4 weeks and only device-related AEs and serious adverse events (SAEs) thereafter. SAEs, defined as events that are fatal, life-threatening, require hospitalization, or result in disability, will be reported promptly and managed according to institutional regulations. Potential risks include local reactions at the implantation site, delayed healing, and general perioperative complications such as bleeding, infection, nerve injury, and thrombosis. All procedures will be performed using standard sterile techniques to minimize risk, and the study will be suspended if significant safety concerns arise.

## 3. Discussion

This study aims to evaluate the efficacy and safety of arthroscopy-assisted core decompression combined with OCP/Gel implantation for osteonecrosis of the femoral head (ONFH). This combined approach integrates mechanical support, biological regeneration, and intra-articular assessment, representing a minimally invasive joint-preserving strategy. This approach may be particularly beneficial in patients with pre-collapse or early-stage ONFH, especially in those with medium-to-large necrotic lesions at risk of collapse.

Although core decompression is widely used for early-stage ONFH, its therapeutic efficacy as a standalone procedure remains limited and controversial [10,11]. Previous studies have highlighted that conventional joint-preserving procedures may not adequately prevent disease progression, particularly in patients with larger lesions. Therefore, additional strategies to enhance both structural stability and biological repair are required. Artificial bone grafting may address these limitations by providing mechanical support to the necrotic area, thereby improving structural integrity and reducing the risk of femoral head collapse [27,28]. In addition, osteoconductive materials such as OCP/Gel may facilitate bone regeneration within the necrotic lesion. Compared with conventional bone substitutes, OCP/Gel has demonstrated superior osteoconductivity [31,32,33,34,35], suggesting its potential to enhance bone remodeling and repair. Various osteoconductive materials, including β-tricalcium phosphate, hydroxyapatite, and allogeneic bone grafts, have been used in conjunction with core decompression. OCP/Gel may provide additional biological advantages because of its biodegradability and pro-angiogenic properties, although comparative clinical evidence remains limited. By providing structural support to the subchondral bone and promoting bone remodeling within the necrotic area, this approach may help delay or prevent femoral head collapse. Furthermore, the use of hip arthroscopy offers an important advantage in this study [38,39]. Arthroscopy enables direct visualization of intra-articular structures and allows for the simultaneous assessment and treatment of concomitant pathologies, such as labral tears and cartilage damage. Addressing these intra-articular factors may contribute to improved clinical outcomes, as hip joint dysfunction in ONFH is influenced not only by intraosseous pathology but also by intra-articular abnormalities. In addition, arthroscopy may improve the accuracy of graft placement and reduce the risk of intra-articular complications.

The concept of combining mechanical and biological strategies is consistent with recent trends in orthopedic research. Integrated approaches targeting multiple aspects of disease pathophysiology may provide greater therapeutic benefit than single-modality treatments [17,18,19,20,21,22,23,24,25,26,27]. Previous studies support the importance of combining different therapeutic mechanisms to improve clinical outcomes. In this context, the present study introduces a novel treatment strategy that combines core decompression, osteoconductive biomaterials, and arthroscopic techniques.

This study has several strengths. It evaluates a novel combined technique that addresses both intraosseous and intra-articular pathology. In addition, the externally controlled design with propensity score matching allows for adjustment of baseline differences between groups, improving the validity of comparisons. Furthermore, both radiographic and clinical outcomes are assessed, enabling a comprehensive evaluation of treatment effectiveness. However, several limitations should be acknowledged. First, this is a single-center study with a relatively small sample size, as this trial was designed as a pilot study, which may limit generalizability. Second, the use of a historical control group introduces potential selection bias and residual confounding, despite statistical adjustment. In particular, differences in treatment indications and clinical practice over time may have influenced the outcomes. Baseline is defined as the time of surgery in the intervention group and the time of initial diagnosis in the historical control group. This asymmetry may introduce bias because disease progression occurring before surgery in the intervention group is not captured. Furthermore, residual confounding due to unmeasured factors, including corticosteroid use, alcohol consumption, symptom duration, and temporal changes in clinical practice, cannot be completely excluded. In addition, baseline patient-reported outcome measures, including the HHS and JHEQ, are not available for the historical control cohort. Therefore, these outcomes will be evaluated descriptively within the intervention group, and direct comparisons between groups will not be possible. Third, the follow-up period is limited to short-term outcomes, and long-term durability, including prevention of femoral head collapse and conversion to total hip arthroplasty, remains unclear. In addition, the collapse length measurement used in this study has not been formally validated, and intra- and inter-observer reliability were not assessed. Therefore, measurement variability may influence the reproducibility of radiographic outcomes. Finally, variability in surgical technique and patient characteristics may influence the results. Therefore, caution is required when generalizing the findings to other populations and clinical settings. In conclusion, this study is expected to provide important preliminary evidence regarding the feasibility and potential effectiveness of arthroscopy-assisted core decompression combined with OCP/Gel implantation. The findings may contribute to the development of improved joint-preserving strategies for ONFH and support future large-scale prospective studies. This approach may offer a practical and scalable alternative to more complex regenerative therapies.

## Figures and Tables

**Table 1:** Schedule of enrollment, interventions, and assessments (SPIRIT).

	Outpatient Evaluation Before Intervention			In-Hospital	Outpatient/In-Hospital Evaluation					
	Obtain consent	Screening	Enrollment	Intervention	1 week	4 week	3 months	6 months	1 year	discontinuation
Allowance duration	−2 months to screening	−2 months ± 2 week	Screening to day before intervention	± 0 day	± 1 days	± 1 week	± 2 week	± 2 week	± 2 week	―
Obtain consent	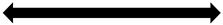									
Research participant background ^a^		●								
Enrollment			●							
Intervention				●						
X-rays		●		●	●	●	●	●	●	●
CT		●					●		●	●
Vital check				●	●					
Blood test ^b^		●		●	●					
Questionnaire			○				○		○	○
Adverse events ^c^				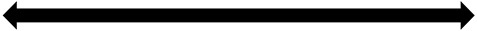						
Physical therapy ^d^					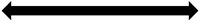					●
Concomitant medication ^e^			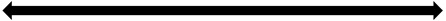							●

● indicates a medically required item, and ○ indicates a required item added for research purposes. a: Age, sex, height, weight, medical history, and comorbidities. This information is collected from the medical record. b: White blood cell count, red blood cell count, Hb, Ht, PLT, TP, ALB, T-BIL, AST, ALT, BUN, Cre, Na, K, Cl, LDH, ALP, CRP. c: Adverse events refer to all undesirable occurrences, such as side effects, and do not necessarily have a causal relationship with the research. d: During hospitalization, the procedure will be performed once a day. After discharge, depending on the patient’s ability to bear weight, it will be performed approximately once a week for the first six weeks post-surgery, and if necessary, approximately three months after discharge. e: Data will be collected only for steroids, immunosuppressants, and osteoporosis medications, from registration to the end of treatment. ➡ indicates the sequence or progression from one study phase/time point to the next.

## Data Availability

No new data were created in this study. Data sharing is not applicable to this article.

## Abbreviations

The following abbreviations are used in this manuscript:

ONFH	Osteonecrosis of the femoral head
THA	Total hip arthroplasty
BMP	bone morphogenetic proteins
β-TCP	β-tricalcium phosphate
FGF	fibroblast growth factor
OCP/Gel	Octacalcium phosphate/gelatin composite
jRCT	Japan Registry of Clinical Trial
JIC	Japanese Investigation Committee
HHS	Harris Hip Score
JHEQ	Japanese Orthopaedic Association Hip-Disease Evaluation Questionnaire
CT	computed tomography
AEs	Adverse events
SAEs	serious adverse events
